# Molecular genetic variability in coenopopulations of Caragana jubata (Pall.) Poir. (Fabaceae) in the mountains of Central Asia and Southern Siberia revealed using ISSR-markers

**DOI:** 10.18699/vjgb-26-22

**Published:** 2026-04

**Authors:** D.A. Krivenko, O.A. Chernysheva, I.N. Kuban, E.V. Zhmud, I.V. Gorbenko, A.A. Achimova, O.Yu. Vasilyeva, O.V. Dorogina

**Affiliations:** Siberian Institute of Plant Physiology and Biochemistry of the Siberian Branch of the Russian Academy of Sciences, Irkutsk, Russia; Siberian Institute of Plant Physiology and Biochemistry of the Siberian Branch of the Russian Academy of Sciences, Irkutsk, Russia; Central Siberian Botanical Garden of the Siberian Branch of the Russian Academy of Sciences, Novosibirsk, Russia; Central Siberian Botanical Garden of the Siberian Branch of the Russian Academy of Sciences, Novosibirsk, Russia; Siberian Institute of Plant Physiology and Biochemistry of the Siberian Branch of the Russian Academy of Sciences, Irkutsk, Russia; Altai Branch of the Central Siberian Botanical Garden of the Siberian Branch of the Russian Academy of Sciences “Gorno-Altaian Botanical Garden”, Kamlak, Republic; Central Siberian Botanical Garden of the Siberian Branch of the Russian Academy of Sciences, Novosibirsk, Russia; Central Siberian Botanical Garden of the Siberian Branch of the Russian Academy of Sciences, Novosibirsk, Russi Novosibirsk State University, Novosibirsk, Russia

**Keywords:** camel’s tail caragana, vulnerable species, coenopopulations, genetic polymorphism, genetic diversity parameters, карагана гривастая, уязвимый вид, ценопопуляции, генетический полиморфизм, параметры генетического разнообразия

## Abstract

The genetic structure was studied using five ISSR markers in 44 individuals of samples obtained from eight coenopopulations (CPs) of Caragana jubata (Pall.) Poir. in the mountainous conditions of Central Asia – in the Western Pamirs and Inner Tien Shan (Kyrgyzstan) and in Southern Siberia – in Altai (Republic of Altai), Western (Republic of Tyva) and Eastern (Republic of Buryatia) Sayan. We studied the species in a range of geographical distances of more than 2,500 km and in the range of absolute altitudes of 1,570–3,680 m. Caragana jubata is listed in the Red Data Books of eight subjects of the Russian Federation. The species population is declining, including due to anthropogenic impact. The aim of the current work is to identify genetic diversity and heterogeneity in C. jubata coenopopulations depending on their geographic and altitudinal confinement in the mountains of Central Asia and Southern Siberia. It was shown that in undisturbed locations, the studied CPs of this species were characterized by a high number of individuals and by the occupied area of more than 100 m2. Almost every sample from the C. jubata CP studied by us (except for representatives from CP7) contained genotypes possessing unique DNA fragments. The highest proportion of such genotypes (75 %) was found in the sample from CP3 (Inner Tien Shan, Teskey-Ala-Too Ridge, absolute altitude 2,550 m). We did not find unique fragments in the genotypes of individuals from the studied sample of CP7 (Western Sayan, Republic of Tuva). Anthropogenic impact on plants at this location is a possible reason for that. The revealed predominance of intrapopulation genetic variability over interpopulation genetic variability in samples from eight C. jubata CPs studied by us may indicate the stability of representatives of this species within the parts of the range studied by us.

## Abstract (ru)

Исследована генетическая структура по пяти ISSR-маркерам у 44 особей в выборках из восьми ценопопуляций (ЦП) Caragana jubata (Pall.) Poir. в горных условиях Центральной Азии – на Западном Памире и Внутреннем Тянь-Шане (Кыргызстан) и в Южной Сибири – на Алтае (Республика Алтай), Западном (Республика Тыва) и Восточном (Республика Бурятия) Саяне. Вид изучен в диапазоне географических расстояний, составляющих более 2.5 тыс. км и в диапазоне абсолютных высот 1570–3680 м. Caragana jubata занесена в Красные книги восьми субъектов Российской Федерации. Цель настоящей работы – выявление генетического разнообразия и гетерогенности в ЦП C. jubata в зависимости от их географической и высотной приуроченности в горах Центральной Азии и Южной Сибири. Исследования показали, что в ненарушенных местонахождениях изученные ЦП этого вида характеризовались высокими параметрами численности особей и занимали площадь более 100 м2. Практически в каждой изученной нами выборке из ЦП C. jubata (кроме представителей из ЦП7) обнаружено наличие генотипов, содержащих уникальные фрагменты ДНК. Наибольшая доля (75 %) таких генотипов установлена в выборке из ЦП3 (Внутренний Тянь-Шань, хр. Тескей-Ала-Тоо, абсолютная высота 2550 м). Нами не найдено уникальных фрагментов в генотипах у особей из исследованной выборки в ЦП7 (Западный Саян, Республика Тыва). Возможная причина – антропогенное воздействие на растения в данном местонахождении. Выявленное преобладание внутрипопуляционной генетической изменчивости над межпопуляционной в выборках из восьми изученных ЦП C. jubata может свидетельствовать об устойчивости представителей этого вида в пределах исследованных нами частей ареала.

## Introduction

Caragana jubata (Pall.) Poir. (camel’s tail caragana) is a Neogene (Miocene-Pliocene) relict that diverged from its closest relatives more than 4.8 million years ago (Zhang M.-L. et al., 2016). It is a thorny monoecious shrub with bisexual flowers, 0.3–2.5 m tall. It is a heliophilous, predominantly high-mountain species, a mesopsychrophyte that grows in the forest (from 1,100 to 3,000 m above sea level – on the Stanovoy Highlands and Tien Shan, respectively) and high-mountain belts (up to 4,700 m above sea level – on the Tibetan Plateau), in the Arctic tundra (up to 500 m above sea level – in the Kharaulakh Mountains) on rocky placers and cliffs, along the banks of rivers, under the canopy of sparse forests (Koropachinsky, Vstovskaya, 2012; Churyulina, 2021; Oorzhak et al., 2024). The modern range of this relict species is Pan-Asian and disjunctive: from the Tibetan Plateau, the Himalayas, Tien Shan, Altai, Sayan, and Stanovoy Highlands to the Kharaulakh Mountains and the Kolyma Highlands (Churiulina, Bocharnikov, 2019). Optimal conditions are found in the upper part of the spruce forest belt in the Central Tien Shan, where the plants are well-foliated, bear fruit abundantly, and reach a height of 2.0–2.5 m (Lysova, 1967).

C. jubata is listed in the Red Data Books of eight constituent entities of the Russian Federation. In the Red Data Books of the Republic of Altai (2017), Magadan Oblast (2019), and Krasnoyarsk Territory (2022), it is assigned rarity status category 3 (R) – a rare species with a relict disjunctive range. In the Republics of Buryatia (2023) and Sakha (Yakutia) (2017), Irkutsk Oblast (2020), Trans-Baikal (2017), and Khabarovsk (2019) Territories, it is assigned rarity status category 2 (VU) – a species declining in numbers, including as a result of overexploitation by humans

Limiting factors for the spread of C. jubata may include the isolation of populations from each other and their low abundance, as well as the demand for the basic composition of rocks due to growth primarily on limestones and carbonate sandstones. Furthermore, exogenous geomorphological processes, such as landslides and floods, pose a threat to populations of this species (Semenova, 2007; Koropachinsky, Vstovskaya, 2012). C. jubata is widely used in folk medicine in Eastern Siberia, which leads to unsystematic harvesting of raw materials and the felling of shrubs for medicinal purposes, since harvesting is carried out year-round and plants are harvested whole, including the root system. Livestock grazing also leads to the depletion of natural populations (Semenova, 2007; Parygin, Khudonogova, 2017; Churyulina et al., 2020).

According to the study of the composition of biologically active substances and the pharmacological activity of an aqueous extract of aerial plant parts, the dominant fraction of biologically active substances in C. jubata are polyphenolic compounds (flavonoids), primarily mono- and diglycosides, which are derivatives of O- hydroxylated flavonols. The antioxidant activity of C. jubata aqueous extracts in vitro, their hepatoprotective, antiviral, anti-inflammatory and wound-healing activity were revealed, while the absence of toxicity was noted. Therefore, aqueous extracts are recommended for the treatment of skin and mucous membrane diseases associated with bacterial inflammatory processes (Kakorin et al., 2020).

Decoctions and alcoholic extracts of the aerial parts of C. jubata were found to possess hepatoprotective, antitumor, anti-inflammatory, and antiviral properties (Kakorin et al., 2018a, b). An ethanol extract of C. jubata is used in traditional Chinese medicine to treat hypertension and, in an experiment on rats, reduced infarct size, reduced cerebral edema, and improved neurological parameters in induced ischemic stroke (Wang et al., 2020; Zhao et al., 2023). Methanol extracts of C. jubata roots exhibited cytotoxic activity against hepatocellular carcinoma cell lines (Yang et al., 2015).

Studies of the genetic structure of C. jubata populations are extremely limited. For example, intraspecific differentiation and phylogeography of this species were studied using sequencing studies of nuclear and chloroplast markers in samples from Tibet, Tien Shan, Eastern Sayan, and the northeastern coast of the Sea of Okhotsk (Zhang et al., 2016; Hantemirova et al., 2024). Using SSR markers, the effect of altitudinal gradients on the genetic structure of alpine C. jubata plants was studied (Yao et al., 2025). We found no literature sources on the study of patterns of variability for ISSR markers in CPs from geographically distant locations at different absolute altitudes, which affect the genetic differentiation of C. jubata CPs.

In a review by T. Ohsawa and I. Yuji (2008), they summarized data on the influence of absolute elevations of herbaceous perennials and dendroflora on intrapopulation polymorphism using neutral molecular markers at regional scales (approximately 1–10 km). Larger scales (>102 km) were not considered, since, according to the authors, large horizontal distances influence genetic variability no less than limited vertical distances. Isoenzymes, RAPD, or SSR were used to assess genetic polymorphism. According to the authors’ conclusions, a specific locus or allele at a given locus can exhibit clear differentiation between populations at different elevations as a result of natural selection.

It was shown that in studies of the genetic heterogeneity of coenopopulations (CPs) of different plant species along altitudinal gradients, the genetic diversity within a CP varied according to one of four patterns of intrapopulation genetic variability, usually represented by expected heterozygosity: L < M > H, L > M > H, L <M <H, or L = M = H, where L, M, and H represent low, medium, and high altitudes, respectively. In particular, a number of studies (Oyama et al., 1993; Yifru et al., 2006) have shown that CPs at intermediate altitudes had greater genetic diversity than those in lower and higher habitats, since geographically central populations, according to the authors, are in optimal environmental conditions, while peripheral populations are in suboptimal conditions. For example, in Arabis serrata Franch. & Sav. (cressweed) on an absolute altitude gradient of 1,440–2,400 m and Triticum turgidum subsp. durum (Desf.) Husn. (durum wheat) – 1,500–3,300 m, the highest intrapopulation variability was observed in middle parts of their altitudinal ranges, respectively, examined with enzyme loci and 29 nSSR loci (Oyama et al., 1993; Yifru et al., 2006).

X. Zhang et al. (2006) found that highly heterogeneous environmental conditions imposed by elevational gradients can influence neutral regions closely associated with the genomic region under selection. Even in cases where differentiation is not detected, intrapopulation genetic variability often changes along elevational gradients.

Identification of the status of genetic variation depending on absolute altitude will help to determine future research directions. In particular, the effectiveness of selection of the most resistant forms of C. jubata during introduction to cultivation will be significantly increased by using propagation material from individuals from the most genetically heterogeneous CPs. Therefore, it is crucial to study the genetic diversity and population structure of wild vulnerable plant species to ensure their most rational use (Liu et al., 2024; Yao et al., 2025).

The aim of the current study was to identify genetic diversity and heterogeneity in C. jubata coenopopulations depending on their geographic and altitudinal distribution in the mountains of Central Asia and Southern Siberia.

## Materials and methods

**Fig. 1.**
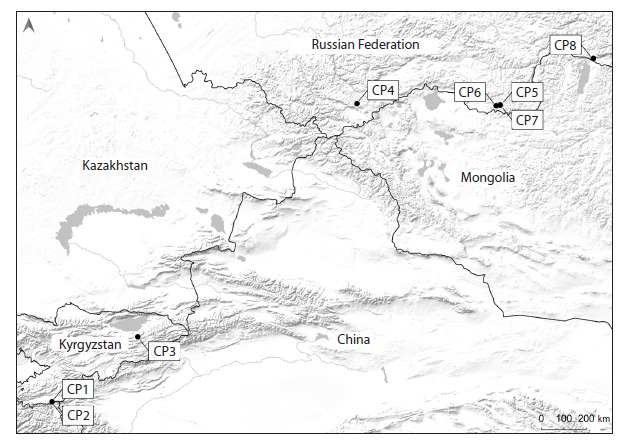
Locations of the studied specimens of C. jubata in Kyrgyzstan (CP1–CP3) and the Russian Federation: in the Republics of Altai (CP4), Tyva (CP5–CP7) and Buryatia (CP8).

We collected the research material in 2015 and 2023– 2024 in the central areas of five regions located in mountainous conditions: in Kyrgyzstan (Western Pamir – CP1, CP2 and Inner Tien Shan – CP3) and in three constituent entities of the Russian Federation (Southern Siberia): in the Republics of Altai (CP4), Tyva (CP5–CP7) and Buryatia (CP8) at altitudes of 1,575–3,680 m above sea level (Fig. 1, Supplementary Material 1)

Supplementary Materials are available in the online version of the paper: https://vavilov.elpub.ru/jour/manager/files/Suppl_Kriv_Engl_30_2.pdf


For the genetic analysis, leaves from 5–6 individuals in each CP were collected. DNA was isolated from the leaves using a standard CTAB-based method (Nabieva et al., 2020). PCR was performed on a C1000 amplifier (Bio-Rad, USA) in a volume of 25 μl. The reaction mixture contained: 1.5 units of Taq DNA polymerase (Medigen, Russia); 2.7 mM of MgCl2; 0.8 mM of ISSR primer (Medigen, Russia); 2 μl of DNA solution; 2 μl of mQ H2O. The amplification program consisted of a DNA denaturation step for 90 s at 94 °C and 35 cycles, each of which included 40 s at 94 °C, 45 s of primer annealing, and 90 s at 72 °C. The final step of nucleotide chain elongation lasted 5 min at 72 °C.

Electrophoretic separation of the amplification products was performed in 1.5 % agarose gel in 1x TAE buffer at a voltage of 4 V/cm. To estimate the length of the PCR products, the DNA marker 100 bp DNA Ladder (no stain) (BIORON GmbH, Germany) was used.

**Fig. 2.**
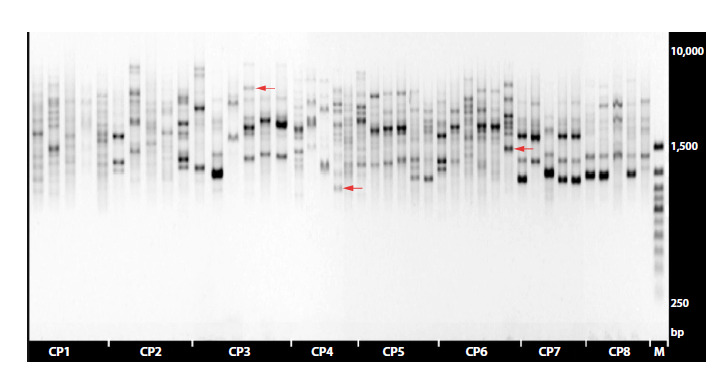
Electropherogram of DNA fragment amplification products with the UBC857 primer. Samples taken from C. jubata CPs CP1–CP8 – CP numbers; M – the molecular weight marker; arrows indicate unique fragments of genotypes.

In total, we tested five ISSR primers that showed the highest efficiency in working with rare plant species (Dorogina et al., 2023; Zhmud et al., 2023, 2024): HB12 ((CAC)3GC), UBC834 ((AG)8YT), UBC856 ((AC)8YA), UBC857 ((AC)8YG) and UBC881 ((GGTG)3). Of these, UBC857 was selected, as it turned out to be the most informative (Fig. 2, Supplementary Material 2).

The primary analysis of the study results was performed via GenAlEx6.1, a software package and specialized macro for MS-Excel (Smouse et al., 2017). Further analysis and results visualization were performed using the R programming environment (R Core Team, 2024). Data preparation was conducted using the ‘tidyverse’ package (Wickham et al., 2019). The Hamming distances and the UPGMA method were used to construct the dendrogram, and the tree was visualized using the ‘ggtree’ package (Xu et al., 2022). Correspondence analysis was performed using the ‘adegenet’ package (Jombart et al., 2008) and the result was used for additional visualization of the genetic structure of the studied CPs. The correspondence analysis, similar to principal component analysis, allows for data dimensionality reduction and visualization; however, it is designed for categorical data and uses chi-squared distance, which is more suitable for genetic data, where relative allele frequencies are important.

Isolation by distance analysis was performed according to the method described by L.S. Nørgaard et al. (2017), and isolation by elevation analysis was performed according to the method of M.-M. Shi et al. (2011). Both analyses were performed using the ‘adegenet’ package (Jombart et al., 2008), with Jaccard distances used as genetic distances; the resulting plots were generated using the ‘ggpubr’ package (Kassambara, 2023). Isolation by distance is a phenomenon that describes patterns of genetic variation in populations arising due to spatially restricted gene flow and is defined as a decrease in the genetic similarity between populations with increasing geographic distances between them (Wright, 1974). Isolation by elevation is a similar phenomenon but with elevation differences between populations used as a spatial component

## Results

Our studies have shown that the C. jubata CPs we studied are characterized by high individual abundances and occupy areas exceeding 100 m2 (Supplementary Material 1). More than 200 individuals were identified in CP1 and CP2, whereas in CP3–CP6, 20 to 50 individuals were found. The individuals inhabited in general undisturbed sites with high grass cover that exceed 50 %. CP7 was an exception, as its few individuals (approximately 10 specimens) were subject to anthropogenic impact due to the construction of a road through its location. In this CP, the aboveground parts of the individuals appeared damaged and lacked developed reproductive organs. The grass cover in this location was also low (Supplementary Material 1).

Molecular genetic studies in samples from the CPs of C. jubata showed that the number of genotypes containing unique fragments in the studied sample from all CPs was 18, and the number of repeated variants was nine (Table 1). In each of the seven studied CPs (CP1–CP6, CP8), except CP7, genotypes with unique fragments were identified that were not observed in individuals from the other studied CPs (Table 1). In the sample of individuals from CP7, only two genotype variants identical to the genotype variants of individuals 4.3 and 8.2 (respectively, from CP4 and CP8) were identified (Table 1, Fig. 2).

**Table 1.**
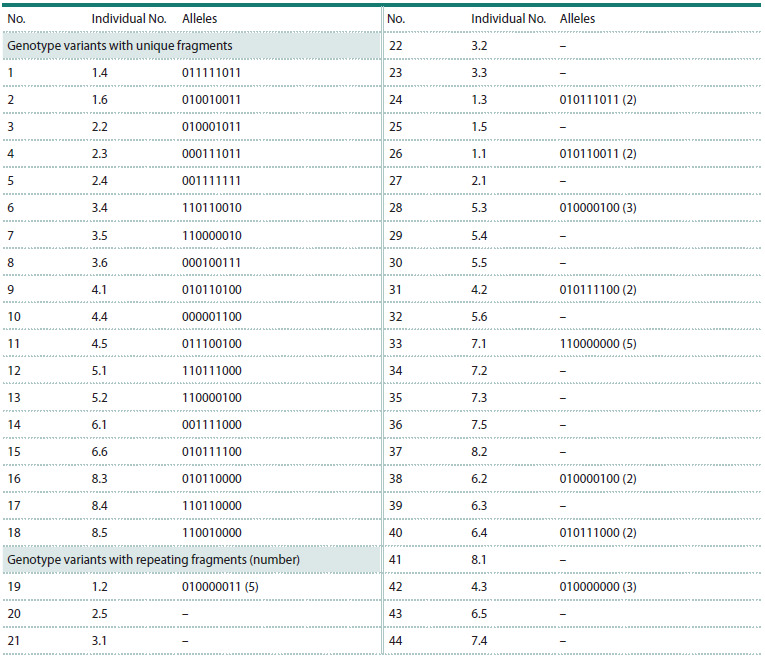
Alleles of genotypes with repetitive and unique fragments in individuals from the studied CPs of C. jubata Note. 1 – presence of an allele, 0 – absence.

The number of genotype variants in C. jubata individuals from the studied CPs varied from 2 to 5 (Table 2). The proportion of genotypes with unique fragments in the studied samples of the CPs varied from none (in CP7) to 75 % (in CP3) (Table 2, Fig. 2). It was calculated as the ratio of the number of unique genotypes containing unique fragments to the total number of genotype variants in each CP. Representatives from CP3 grew in high-mountain conditions at an absolute altitude of 2,550 m, i. e. they occupied an intermediate geographic and altitudinal position between the Pamir and South Siberian CPs (Supplementary Material 1)

**Table 2.**
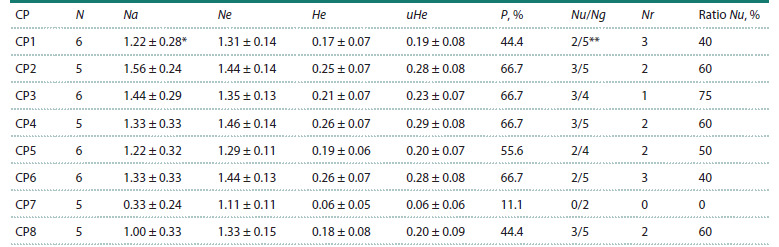
Parameters of genetic diversity in samples from C. jubata CPs * Stands for mean value ± standard error; N – number of samples; Na – number of alleles; Ne – number of effective alleles; He – expected heterozygosity; uHe – objective expected heterozygosity; P – proportion of polymorphic loci; Nu/Ng – ratio of the number of genotypes with unique fragments to the number of genotype variants in the CP; Nr – number of repeating genotype variants in each CP. ** The denominator indicates the total number of genotype variants in the samples from the CP.

**Fig. 3.**
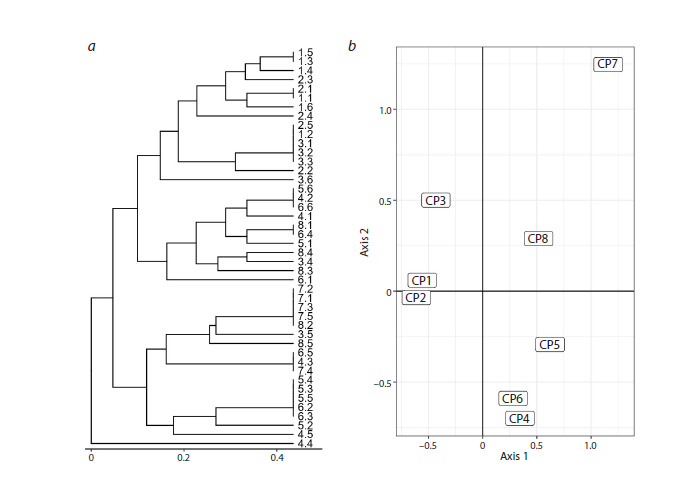
The clustering of C. jubata CPs into groups based on the similarity of genetic structure. a – UPGMA dendrogram constructed using the ISSR spectra data obtained with the UBC857 primer; b – the result of correspondence analysis. CP1 – individuals 1.1–1.6; CP2 – individuals 2.1–2.5; CP3 – 3.1–3.6; CP4 – individuals 4.1–4.5; CP5 – individuals 5.1–5.6; CP6 – individuals 6.1–6.6; CP7 – individuals 7.1–7.5; CP8 – individuals 8.1–8.5.

According to cluster analysis, the presence of two particularly genetically distant groups of individuals was revealed. These are samples from CP1–CP2 and CP5– CP8. They are characterized by low genetic similarity, as shown in the distribution diagram of C. jubata individuals according to their genotypic characteristics (Fig. 3), and are located primarily in different clades. Based on genotypic similarity, the studied C. jubata individuals were divided into two main clusters that are in general in agreement with their geographic distribution (Fig. 3a).

According to cluster analysis, the upper part of the tree is predominantly composed of individuals from the mountainous regions of the Western Pamirs and Inner Tien Shan (CP1–CP3). This group also includes some southern Siberian individuals from samples of CP4–CP6 and CP8. The lower part of the tree is generally characterized by a distinct distribution of southern Siberian individuals (CP4–CP8). Individuals from CP7 are located separately, and the closest groups to them are those from southern Siberian CPs. The results of the correspondence analysis also support this distribution (Fig. 3b).

**Fig. 4.**
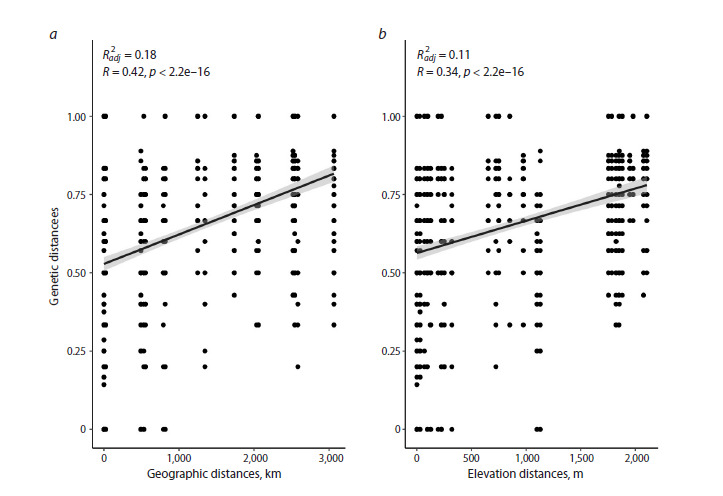
Correlations of genetic distances of C. jubata CPs with geographic distances (a) and relative elevations (b) of their locations. Each point represents a comparison of two individuals, characterized by genetic distance (Y) and geographic/elevation distance (X).

According to the obtained data, individuals in the geographically extreme CPs grew at distances of over 2,500 km from each other. Statistical analysis of the data in the samples from the studied CPs revealed significant correlations between genetic distances and both geographic and elevational distances, indicating isolation by distance and isolation by elevation. That is, as the geographic and elevational distances in the samples of individuals from these CPs increase, so do their genetic distances (Fig. 4).

## Discussion

Genome sequencing data for representatives of the genus Caragana Fabr. revealed that its center of origin is located on the Tibetan Plateau (Zhang M.-L. et al., 2016). During the xerothermic period of the Pleistocene, C. jubata penetrated through mountain systems to the west and north. This is supported, in particular, by data on its modern distribution, which is associated with mountainous regions: the eastern part of the Tibetan Plateau, the northeastern Himalayas, the Central and Southern Tien Shan, the Sayan Mountains, and the Stanovoye Highlands (Churiulina, Bocharnikov, 2019).

We studied C. jubata in samples from the Central Plain, also growing over a wide geographic range. Their locations are situated in five mountainous regions of Central Asia – the Western Pamirs, the Inner Tien Shan, and in southern Siberia – the Altai, Western and Eastern Sayan. C. jubata CPs were studied across an elevation gradient of more than 2,000 m (Supplementary Material 1) and geographic distances of more than 2,500 km. This wide geographic and altitudinal range of C. jubata distribution is consistent with the current understanding that C. jubata species exhibits high ecological heterogeneity (Talovina, 2019).

Within the studied altitudinal range of over 2,000 meters and distances of 2,500 kilometers, we identified 18 genotype variants with unique fragments in samples from 44 representatives of this species, a relatively high number for a rare plant species. The value of genetic diversity lies in the fact that a gene variation that is neutral today can become adaptive later, when environmental conditions change (de Lafontaine et al., 2018). When environmental conditions change, a population with higher genetic diversity is more likely to adapt to the new environment than a population with less diversity. Thus, maintaining a species’ genetic diversity can significantly increase its chances of long-term survival (Chung et al., 2023).

In mountainous environments, plant populations growing at high altitudes demonstrate later flowering and have a shorter flowering period compared to populations of the same species growing at lower altitudes (Reisch et al., 2005). A simple explanation for differences in genetic variation among populations located at the same altitude but separated by mountainous terrain is the difficulty of their diaspore dispersal (Taberlet et al., 1998). A study using paleobotanical and genetic data for Fagus sylvatica L. (European beech) showed that mountain ranges in Europe were not a geographic barrier for this species, but rather facilitated its dispersal. On the contrary, its dispersal was hindered by the presence of large plains or valleys (Magri et al., 2006). From this, the authors concluded that flat landscapes can act as a barrier to the dispersal of diaspores, including those of high-altitude species.

However, rare and endemic plant species are typically characterized by significantly lower genetic diversity and, consequently, higher homozygosity compared to non-endemic species (Hamabata et al., 2019). Genetic homogenization of populations, especially in rare plant species, results in reduced heterozygosity among their individuals. Populations lacking genetic variability are unable to evolve in response to changing environmental conditions and, therefore, may face an increased risk of extinction (Dorogina, Zhmud, 2020).

In addition to genotypes with unique fragments, we identified nine recurring genotype variants in C. jubata specimens taken from the studied CPs. Specimens from CP7 exhibited genotype variants similar to those in specimens from other CPs. The absence of unique genotype fragments in the sample from this CP may be explained by anthropogenic impacts on its specimens in the area where a road is being constructed. We observed a similar situation, for example, in specimens of rare Siberian species Adonis villosa Ledeb. (downy adonis), where only four genotype variants were identified in 40 specimens studied in six CPs in the Republic of Altai. Road construction was also carried out in its habitats, and damage to plants and soil was observed (Zhmud et al., 2024).

We also found genetic homogenization in the habitat of one of the CPs of the rare species Rhodiola rosea L. (rose rhodiola) in the Republic of Altai. Individuals of this species were damaged by trampling at a summer camp and by grazing by cattle (yaks) (Dorogina et al., 2023). It is possible that the C. jubata individuals remaining in CP7 are more resilient to soil damage, in this case related to construction work. Genetic homogenization of individuals in this CP raises concerns about its sustainability and may eventually lead to its complete extinction

Сluster analysis of the distribution of the most polymorphic components detected via ISSR labeling showed that the studied CPs were divided into different groups according to their genetic structure. We previously noted that the locations of the C. jubata CPs we studied differ by more than 2,500 km at their extreme points. A statistically confirmed trend of increasing genetic distances between representatives from different CPs with increasing spatial distance was found.

We also found a tendency toward an increased proportion of unique genotypes (containing unique fragments) in the C. jubata CP growing in the Tien Shan mountains at an altitude of 2,550 m – the middle part of the studied altitudinal range – compared to the Pamir and South Siberian CPs. According to T. Ohsawa and I. Yuji (2008), such populations may experience more favorable environmental conditions than peripheral populations growing in suboptimal conditions. The authors believe that mountain isolation plays a crucial role in the diversification and evolution of species.

Adaptation of wild plants to stress with increasing altitude is a complex process achieved through both modification and genetic variability (Safaralikhonov, Aknazarov, 2021). Molecular studies by P.K. Bhardwaj et al. (2013) showed that C. jubata exhibits a predominance of genes encoding chaperones and genes involved in growth and development at low temperatures, which are among the main signals acting on plants at high altitudes. According to these studies, such genes are expressed in C. jubata in its natural habitat. Their homologs in Arabidopsis thaliana (L.) Heynh. (Thal’s cress), Glycine max (L.) Merr. (soybean), and Oryza sativa L. (rice) do not exhibit a similar trend in gene expression at low temperatures

Constitutive expression and rapid regulation of the mentioned genes suggest the ability of C. jubata to tune its cellular machinery to support growth and development in its ecological niche. This molecular and physiological plasticity allows C. jubata to thrive, particularly in the cold, high-altitude desert of the Himalayas (Bhardwaj et al., 2013). The trends in genetic differentiation that we identified in the studied C. jubata CPs likely result from the adaptation of this species to various ecological and geographical conditions.

G.P. Semenova (2007) classified C. jubata as a medium- potential species when introduced into the foreststeppe zone of Western Siberia. Such species reproduce by seed or vegetatively, require specific soil composition, irrigation, and sites with specific lighting conditions, and flower and fruit annually. However, they are susceptible to frost, and their reproductive capacity may be reduced in unfavorable years. G.P. Semenova (2007) also classified C. jubata as a perennial plant, as the lifespan of the introduced population in local conditions reached 17 years

The extensive range of this species and relatively high genetic diversity suggest that individuals in samples taken from most of the CPs we studied are stable and can serve as source material for the creation of promising introduction populations

Thus, statistical analysis of genetic variability revealed a significant effect of geographic isolation between genotypes, due to the large distances between the studied C. jubata CPs. The influence of altitudinal isolation on genetic distances between individuals from different CPs was also confirmed.

## Conclusion

We studied C. jubata in samples from eight CPs growing across a wide range of altitudes (over 2,000 m – from 1,575 to 3,680 m) and geographic distances (over 2,500 km) in the mountains of the Pamirs, Tien Shan, and Southern Siberia. ISSR analysis of polymorphic DNA fragments revealed an increasing number of genotypes with unique fragments and the proportion of polymorphic loci in individuals of this species growing in the Tien Shan Mountains at an absolute altitude of 2,550 m, located in the middle of the studied elevation range. We also found a statistically confirmed trend toward increase of genetic distances in samples of representatives from different CPs as geographic distance increase.

The prevalence of intrapopulation genetic variability over interpopulation genetic variability we identified in our studies may indicate the stability of these CPs within the studied parts of this species’ range and their good adaptive capacity. The exception was samples of individuals from CP7, which grew in the Republic of Tyva and were subject to anthropogenic impact, where we did not detect any genotypes with unique fragments.

## Conflict of interest

The authors declare no conflict of interest.
